# Dual contribution of TRPV4 antagonism in the regulatory effect of vasoinhibins on blood-retinal barrier permeability: diabetic milieu makes a difference

**DOI:** 10.1038/s41598-017-13621-8

**Published:** 2017-10-12

**Authors:** David Arredondo Zamarripa, Ramsés Noguez Imm, Ana María Bautista Cortés, Osvaldo Vázquez Ruíz, Michela Bernardini, Alessandra Fiorio Pla, Dimitra Gkika, Natalia Prevarskaya, Fernando López-Casillas, Wolfgang Liedtke, Carmen Clapp, Stéphanie Thébault

**Affiliations:** 1 Instituto de Neurobiología, Universidad Nacional Autónoma de México (UNAM), Campus UNAM-Juriquilla, 76230 Querétaro, Mexico; 2 Univ. Lille, Inserm U1003 - PHYCEL – Physiologie Cellulaire, F-59000 Lille, France; 3 Department of Life Science and Systems Biology, University of Torino, 10123 Torino, Italy; 4 Instituto de Fisiología Celular, UNAM, C.U. Mexico; 5 Department of Medicine and Neurobiology, and Center for Translational Neuroscience, Duke University Medical Center, Durham, NC 27710 USA

## Abstract

Breakdown of the blood-retinal barrier (BRB), as occurs in diabetic retinopathy and other chronic retinal diseases, results in vasogenic edema and neural tissue damage, causing vision loss. Vasoinhibins are N-terminal fragments of prolactin that prevent BRB breakdown during diabetes. They modulate the expression of some transient receptor potential (TRP) family members, yet their role in regulating the TRP vanilloid subtype 4 (TRPV4) remains unknown. TRPV4 is a calcium-permeable channel involved in barrier permeability, which blockade has been shown to prevent and resolve pulmonary edema. We found TRPV4 expression in the endothelium and retinal pigment epithelium (RPE) components of the BRB, and that TRPV4-selective antagonists (RN-1734 and GSK2193874) resolve BRB breakdown in diabetic rats. Using human RPE (ARPE-19) cell monolayers and endothelial cell systems, we further observed that (i) GSK2193874 does not seem to contribute to the regulation of BRB and RPE permeability by vasoinhibins under diabetic or hyperglycemic-mimicking conditions, but that (ii) vasoinhibins can block TRPV4 to maintain BRB and endothelial permeability. Our results provide important insights into the pathogenesis of diabetic retinopathy that will further guide us toward rationally-guided new therapies: synergistic combination of selective TRPV4 blockers and vasoinhibins can be proposed to mitigate diabetes-evoked BRB breakdown.

## Introduction

Diverse conditions, including diabetic retinopathy and macular edema, are associated with exacerbated leakage through the blood-retinal barrier (BRB)^1,2^. The BRB is comprised of inner and outer components that mainly refer to vascular endothelial and retinal pigment epithelial (RPE) cells, respectively^1^. Although high glucose conditions predominantly affect retinal capillaries, the damage to RPE cells has been increasingly recognized to play a major role in the progression of these diseases^3,4^. Nevertheless, its regulation has been less studied than that of retinal capillaries in the context of diabetes. Additionally, that most clinical therapies address symptoms rather than the molecular pathophysiology of diabetic retinopathies^5,6^ indicates that many molecular and cellular mechanisms underlying damage to the BRB by high glucose levels remain to be characterized. More particularly, advances in understanding the key role of endogenous cytokines, their conate receptors and ion channels in BRB regulation may lead to the development of novel therapeutic options for rationally-targeted treatment of diabetic retinopathy and macular edema.

Vasoinhibins, derived from prolactin cleavage, are endogenous regulators of angiogenesis and vascular function that occur naturally in the retina^7^. It has been shown that patients with diabetic retinopathy have lower levels of circulating vasoinhibins than nondiabetic patients^8^. Increasing ocular levels of vasoinhibins were reported to protect against the pathological increase in BRB permeability associated with diabetes^9–12^. Vasoinhibins were recently shown to reduce BRB permeability by targeting both its main inner and outer components^13^; however, their action mechanisms have been best described in vasculature. Vasoinhibins regulate endothelial cell permeability by lowering NO production^10,13,14^ and stabilizing the actin cytoskeleton^13^. Vasoinhibins reduce NO production by limiting endothelial NOS (eNOS) activation through phosphorylation and Ca^2+^/calmodulin binding^15^. Vasoinhibins have been indeed shown to abrogate Ca^2+^ entry through both capacitative^16,17^ and receptor-operated pathways^16^ in endothelial cells. Further evidence supports the idea that vasoinhibins regulate Ca^2+^ homeostasis by interfering with the activity of the Ca^2+^-permeable transient receptor potential (TRP) family members, decreasing the expression of canonical subfamily member 5 protein (TRPC5) mRNA in endothelial cells^16^.

Among the 26 members of the mammalian TRP family, all of which are present in the retina^18^, the vanilloid subfamily member 4 protein (TRPV4) uniquely regulates the capillary endothelial barrier^19^. TRPV4 is a non-selective cation channel permeable to Ca^2+^ that was originally identified as an osmotically activated channel^20–22^, but it is also activated by ligands such as phorbol derivatives^23^. TRPV4 has been demonstrated to participate in both capacitative^24^ and receptor-operated Ca^2+^ entry^25–31^, and Ca^2+^ entry through TRPV4 promotes the formation of Ca^2+^-calmodulin complexes, which can bind to TRPV4 enhancing channel activity^32,33^. Ca^2+^ entry through TRPV4 has been also shown to increase lung endothelial cell permeability by disrupting cell-cell or cell-matrix adhesion^34,35^. A mechanism through which TRPV4 activation evokes the reorganization of actin cytoskeleton that associates with increased permeability may involve NO release^36,37^. Inversely, blockage of TRPV4 channels inhibits eNOS activation by phosphorylation^38^ and mitigates pulmonary edema^39^.

Functional expression of TRPV4 has been reported in retinal mouse capillaries^40,41^ and TRPV4 protein in primary cultures of human fetal RPE^42^. Importantly, in this context we do not know about its expression in adult RPE nor about its participation as a regulator of BRB permeability. We therefore tested this possibility and further postulated that vasoinhibins may regulate BRB permeability by blocking TRPV4. This novel concept is rooted in the fact that vasoinhibins exert effects opposite to the ones induced by TRPV4 activation to regulate capillary endothelial barrier (i.e., intracellular Ca^2+^ rise, eNOS phosphorylation, NO release, cytoskeleton reorganization^36,43–46^).

Here, we identified hitherto under-appreciated TRPV4 retinal distribution and observed that TRPV4 is also expressed by both retinal endothelia and RPE. Given that potent and selective TRPV4 antagonists are available^39,47^, we evaluated the outcome of TRPV4 antagonist intravitreal injection after enhanced BRB breakdown due to experimental diabetes induced by streptozotocin and found that TRPV4 antagonism mitigated BRB breakdown to similar levels than vasoinhibins. We explored the possibility that vasoinhibins and TRPV4 antagonism may synergize to regulate BRB permeability, using ARPE-19 cell monolayers and microvascular endothelial cells in hyperglycemic and normoglycemic conditions, mimicking the diabetic and non-diabetic conditions, respectively. We showed that TRPV4 antagonism and vasoinhibins synergize by activating complementary pathways to counteract the diabetes-like effects on RPE permeability. In addition, vasoinhibins can block the exogenously activated TRPV4 to regulate BRB and endothelial permeability, likely by interfering with the TRPV4/Ca^2+^/NO/cytoskeletal reorganization cascade.

## Results

### TRPV4 is expressed in both the inner and outer BRB, and its pharmacological inhibition resolves the streptozotocin-induced increase of BRB permeability similarly to vasoinhibins in rats

**Figure 1:**
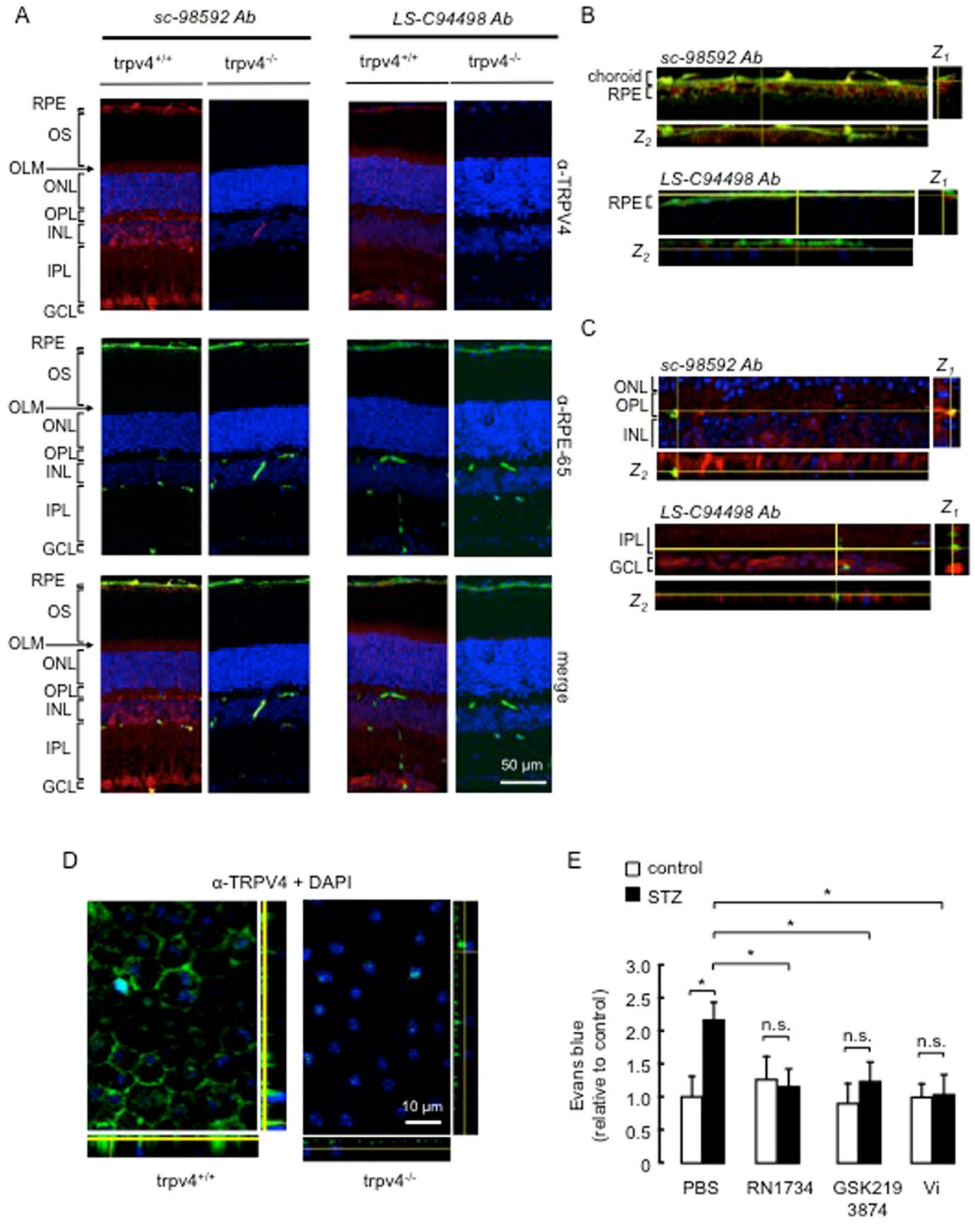
TRPV4 is expressed in both the inner and outer BRB, and its pharmacological inhibition prevents the streptozotocin-induced increase of BRB permeability in rats, similar to the effect of vasoinhibins. (**A**) Representative confocal stack image of transverse sections of *Trpv4* ^+/+^ and *Trpv4* ^−/−^ mouse retinas showing TRPV4 (red), RPE-65 (green), and merge immunofluorescence. The anti-TRPV4 sc-98592 and LS-C94498 antibodies (Ab) were used as indicated and cell nuclei were stained with DAPI. Magnification bars were as indicated. Retinal pigment epithelium (RPE), outer segments (OS), outer nuclear layer (ONL), outer limiting membrane (OLM), outer plexiform layer (OPL), inner nuclear layer (INL), inner plexiform layer (IPL), and ganglion cell layer (GCL). Images were captured in three different regions of the same retina section (n = 3). Both retinas of three animals per group were analyzed. (**B**) Representative confocal image and corresponding projection in z-x (Z_1_, right) and z-y (Z_2_, bottom) of transverse sections of wild-type choroid-RPE (full-size image is shown in supplemental Fig. 3) showing RPE-65 (green) and TRPV4 (red) antibody immunofluorescence. Projections in z correspond to the area indicated by the yellow lines and show that TRPV4 and RPE-65 did not colocalize. (**C**) Representative confocal image and projection in z-x (Z_1_, right) and z-y (Z_2_, bottom) of transverse sections of wild-type OPL and INL (full-size image is shown in supplemental Fig. 3) stained with the anti-TRPV4 sc-98592 or LS-C94498 antibodies (red), the anti-mouse antibody coupled to fluorescein isothiocyanate as a marker of blood vessels (green), as previously reported^49^, and DAPI (blue). (**D**) Representative images of whole mounts of *Trpv4* ^+/+^ and *Trpv4* ^−/−^ mouse RPE stained with the LS-C94498 anti-TRPV4 antibody and DAPI. Projection in z-x (right) and z-y (bottom) is also shown. (**E**) Evaluation of the Evans blue dye content in retinas from control rats intravitreously injected with PBS, the TRPV4 antagonists RN1734 (100 µM) and GSK2193874 (100 nM) or vasoinhibins (Vi, 1 µM) for 24 h and from streptozotocin (STZ)-induced diabetic rats intravitreously injected with PBS, RN1734, GSK2193874 or Vi 24 h before the end of the 4 weeks of diabetes. Values are mean ± s.e.m. normalized to the control (n = 8–14 per group; *P < 0.05). n.s., not significant.

We determined whether the BRB expresses TRPV4 *in situ*. The reliability of TRPV4 detection by the polyclonal sc-98592 antibody was addressed using *Trpv4* ^−/−^ mice^48^ as a negative control, and by comparing the sc-98592 staining to the one obtained with the anti-TRPV4 LS-C94498 antibody, the specificity of which has previously been confirmed^40^. The histology of *Trpv4* ^−/−^ retinas is similar to that of wild-type mice except for the outer limiting membrane that is not visible in *Trpv4* ^−/−^ retinas (supplemental Fig. 1A). Layer thickness was similar in wild-type and *Trpv4* ^−/−^ retinas (supplemental Fig. 1B). Representative images of transversal retinal sections from wild-type mice stained with the sc-98592 antibody showed uniform cytoplasmic immunoreactivity (Fig. 1A). TRPV4 immunostaining was distributed throughout the retina, including cell bodies in the ganglion cell layer; radial processes within the inner plexiform and outer nuclear layers, and outer limiting membrane; cell bodies and capillaries in the inner nuclear layers; and RPE, marked with RPE-65. This immunopositive pattern for TRPV4 was absent in retinal sections from *Trpv4* ^−/−^ mice (Fig. 1A). In parallel, we have repeated the immunohistochemistry on the same tissues using the LS-C94498 antibody (Fig. 1A) and confirmed the previously described immunostaining pattern^40^. Our data further showed a positive staining in the RPE. Projections in z showed that TRPV4 localizes to the basal outer non-pigmented part of the RPE, while RPE-65 is in the cytosol (Fig. 1B). The colocalization of TRPV4 with a fluorescein isothiocyanate, used as blood vessel marker^49^ can be appreciated in Fig. 1C. Immunohistochemistry using the LS-C94498 anti-TRPV4 antibody labeled TRPV4 in whole mounts of mouse RPE, whereas no specific signal appeared in *Trpv4* ^−/−^ RPE (Fig. 1D). These data show that TRPV4 is expressed in both the inner and the outer main component of the BRB.

Given this compelling localization of the channel, the functional role of TRPV4 in the BRB was then investigated. Previous studies have shown that TRPV4 inhibition protects against rupture of the endothelial barrier in the lung^46^ and that expression levels of TRPV4 are altered in both macrovascular^50^ and retinal microvascular^41^ vessels from streptozotocin-induced diabetic rats. Taken together with the fact that BRB breakdown is a feature of diabetes, this information prompted us to examine whether TRPV4 inhibition would eliminate the excessive increase in BRB permeability induced by a diabetic metabolic situation. In the streptozotocin rat preclinical model of diabetes, BRB breakdown occurs as early as 5 days and up to 10 weeks post-streptozotocin treatment^9,11,12,51–55^. Here we confirmed the BRB breakdown in this diabetes model by showing that retinal accumulation of Evans blue-stained albumin doubled at 4 weeks after streptozotocin injection (Fig. 1E). The selective TRPV4-channel blockers, RN1734^56^ and GSK2193874^39^, were injected intravitreally, and 24 h later inhibition of the streptozotocin-induced BRB breakdown was observed (Fig. 1E). In controls without streptozotocin, RN1734 and GSK2193874 alone did not modify BRB permeability (Fig. 1E). As previously reported^10^, vasoinhibins prevented the streptozotocin-induced increase in BRB permeability, and this effect was similar to that of RN1734 and GSK2193874 (Fig. 1E), and vasoinhibins did not modify the basal transport through the BRB (Fig. 1E). Taken together, these data suggest that vasoinhibins may block excessive BRB permeability associated with diabetes by inhibiting TRPV4.

### TRPV4 is expressed in human ARPE-19 monolayers and its protein levels and cellular localization are not altered by exposure to high glucose

**Figure 2:**
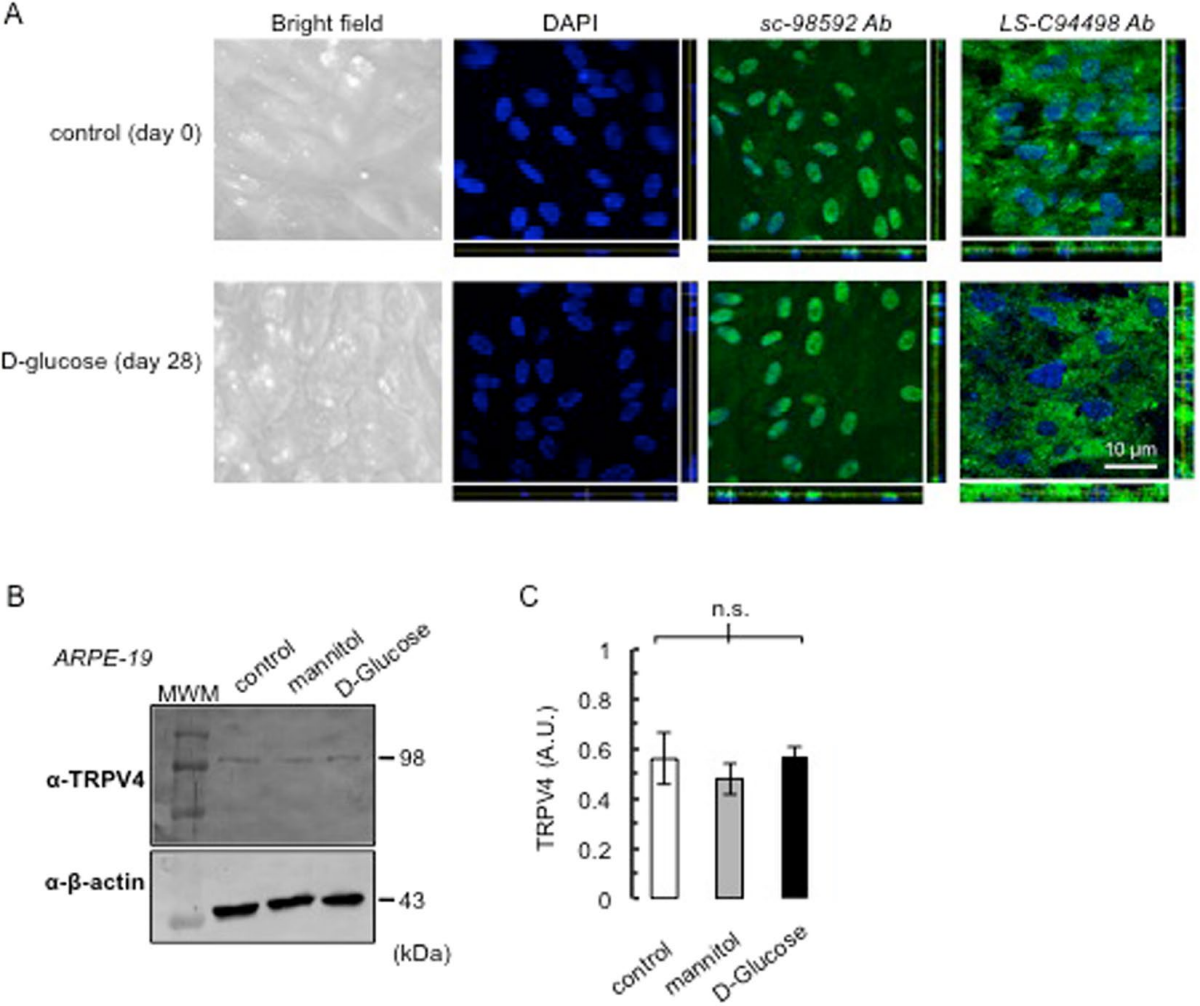
TRPV4 expression in ARPE-19 monolayers. (**A**) Representative phase-contrast microscopy and confocal images of ARPE-19 cultured on Transwell membrane inserts with pore sizes of 0.4 µm (see Methods) stained with DAPI and anti-TRPV4 sc-98592 and LS-C94498 antibodies (Ab), under control conditions (day 0) and after exposure to high glucose (D-glucose) for 28 days. Projection in z-x (right) and z-y (bottom) showed nucleus alignment and TRPV4 localization. Magnification bar was as indicated. (**B**) ARPE-19 cells cultured in 5.5 mM D-glucose (control), 5.5 mM D-glucose plus 19.5 mM mannitol (mannitol), and 25 mM D-glucose (D-glucose) for 28 days were analyzed for TRPV4 protein. Total β-actin served as loading control. Extracts from three independent ARPE-19 cell cultures in each condition were analyzed (N = 3); MWM, molecular weight markers. (**C**) Densitometric analysis of the TRPV4 fragment normalized to β-tubulin (control) expressed in arbitrary units (AU). Values correspond to mean ± s.e.m. for three independent experiments. n.s., not significant.

To study whether vasoinhibins mitigate the streptozotocin-induced increase of BRB permeability by acting directly on the RPE and to test if TRPV4 inhibition mediates this effect, we first provided evidence for monolayer formation and TRPV4 expression in ARPE-19 cells. Phase-contrast microscopy images on cross-sections of ARPE-19 cultures showed that these cells displayed the characteristic features of RPE, including defined cell borders and overall “cobblestone” appearance, and DAPI staining showed that they formed a relatively uniform monolayer (Fig. 2A). Immunocytochemistry showed that TRPV4 is localized throughout the cell (Fig. 2A). Immunolabelling with the sc-98592 antibody showed a pattern resembling the one previously described in cultured capillary endothelial cells^28,41^, while the LS-C94498 antibody preferentially stained cell cytoplasm.

To simulate blood glucose found in the streptozotocin rat model of diabetes, ARPE-19 cells were subjected to basolateral administration of a high glucose concentration (25 mM). After a 28-day exposure to high glucose, ARPE-19 cells conserved defined cell borders and overall “cobblestone” appearance but were flattened, and DAPI staining was indicative of monolayer (Fig. 2A). Projection in z showed that TRPV4 localization was overall similar in ARPE-19 cells exposed or not for 28 days to 25 mM of D-glucose (Fig. 2A). Western blot showed a unique band of 98 kDa (Fig. 2B). We observed that levels of TRPV4 protein were not modified after a 4-week exposure to high glucose in culture (Fig. 2B and C).

### Exposure to high glucose increases and decreases the resistance of ARPE-19 monolayers after 3 and 28 days, respectively

**Figure 3:**
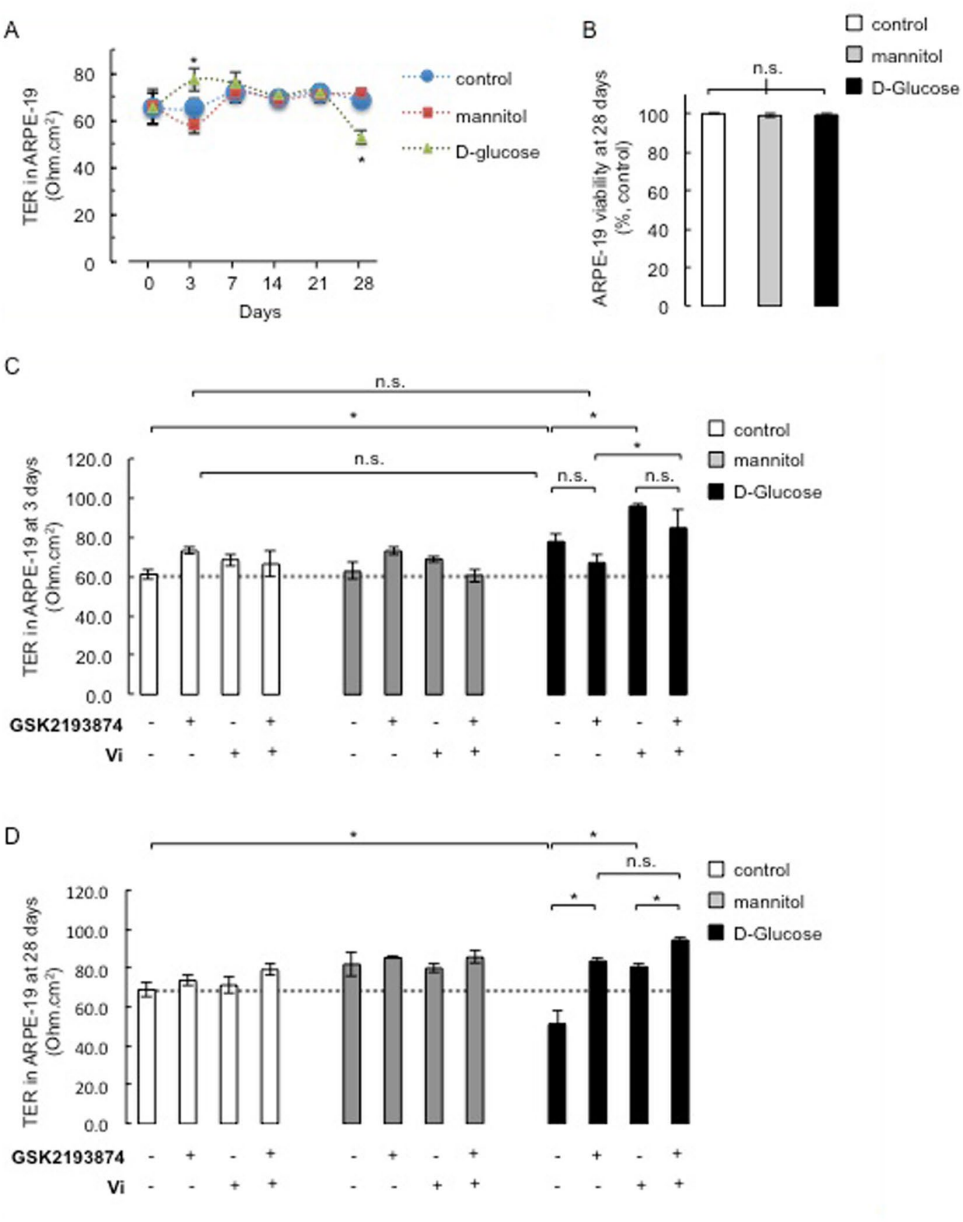
Dual contribution of TRPV4 antagonism and vasoinhibins to regulate the high glucose-induced effects on ARPE-19 monolayer resistance. (**A**) Time course of trans-electrical resistance (TER) in ARPE-19 cell monolayers cultured in 5.5 mM D-glucose (control), 5.5 mM D-glucose plus 19.5 mM mannitol (mannitol), and 25 mM D-glucose (D-glucose) over 28 days. (**B**) Quantification of viability levels in ARPE-19 monolayers treated as previously indicated at day 28. ARPE-19 cells were cultured on inserts with pore sizes of 0.4 µm. TER and MTT signals were normalized to the untreated condition. **(B** and **D**) Quantification of TER values in ARPE-19 monolayers after 3 (**C**) and 28 (**D**) days of culture in control, mannitol, and D-glucose conditions, in the presence and in the absence of GSK2193874 (50 nM), vasoinhibins (Vi, 10 nM) or both. *P < 0.05 from 3 independent experiments. n.s., not significant.

Since inconsistent results were obtained when the trans-electrical resistance (TER) was measured in ARPE-19 cells treated with high glucose concentrations^57–60^, we examined the TER through monolayers of ARPE-19 cells over a period of 4 weeks. Under control conditions, TER was maintained at ∼65 ± 7 Ohm.cm^2^ the whole time (Fig. 3A). The resistance of ARPE-19 monolayers increased after 3 days of exposure to high glucose (Fig. 3A). This increase was transient since TER values at days 0, 7, 14, and 21 were similar in all conditions. At day 28, high glucose associated with a TER decrease, compared with levels under mannitol, which was used as an osmotic control (Fig. 3A). Additionally, some previous studies showed that high glucose conditions associate with cytotoxicity in ARPE-19 cells^61–66^, while others did not^59,67^. We found no change in ARPE-19 cell viability after a 4-week exposure to high glucose (Fig. 3B). Thus, prolonged cultures preserve stable TER and viability in ARPE-19 monolayers, allowing us to explore the effect of TRPV4 antagonism and vasoinhibins on ARPE-19 permeability under hyperglycemic-mimicking conditions.

### TRPV4 antagonism and vasoinhibins activate additive pathways to inhibit the high glucose-related effects on ARPE-19 monolayer resistance

We previously reported that vasoinhibins regulate the RPE barrier in the human ARPE-19 cell line^13,68^. We therefore studied the role of TRPV4 inhibition in this process. GSK2193874 did not prevent the high glucose-induced increase in TER at day 3 (Fig. 3C). Alone or in the presence of mannitol, GSK2193874 did not modify the resistance of ARPE-19 monolayers at day 3 (Fig. 3C). In contrast, vasoinhibins enhanced the high glucose-induced increase in TER after 3 days of culture (Fig. 3C). Vasoinhibins had no effect on the resistance of ARPE-19 monolayers alone or in the presence of mannitol (Fig. 3C). When GSK2193874 was applied concomitantly with vasoinhibins under high glucose, ARPE-19 resistance reached similar values than in the presence of vasoinhibins alone (Fig. 3C). GSK2193874 and vasoinhibin co-administration had no effect on the resistance of ARPE-19 monolayers under control and mannitol conditions.

At day 28, GSK2193874 prevented the high glucose-induced reduction in ARPE-19 TER (Fig. 3D). Vasoinhibins also prevented the high glucose-induced decrease in TER (Fig. 3D). Alone or in the presence of mannitol, neither GSK2193874 nor vasoinhibins modified the resistance of ARPE-19 monolayers (Fig. 3D). When co-administered with vasoinhibins under high glucose, GSK2193874 increased ARPE-19 TER by additional 17% (Fig. 3D).

Taken together, these data show that vasoinhibins regulate ARPE-19 permeability exposed to high glucose through a mechanism that is independent from TRPV4 blockage.

### Vasoinhibins can protect BRB from increased endothelial permeability evoked by TRPV4-activation under non-diabetic conditions

**Figure 4:**
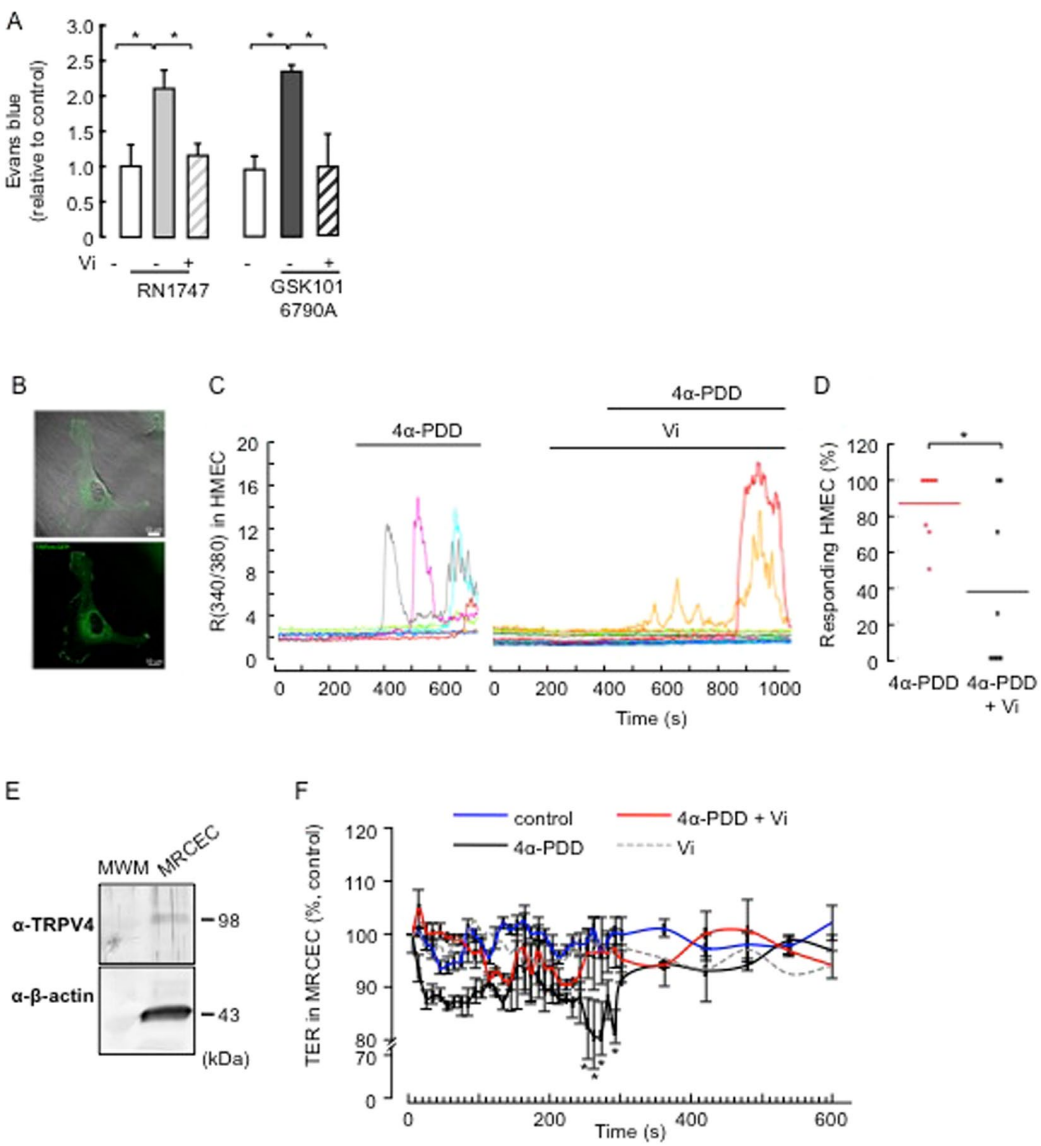
Vasoinhibins block TRPV4-induced BRB breakdown in rat retinas and TRPV4-induced Ca^2+^ transients and TER reduction in endothelial cell systems in endothelial cell systems. (**A**) Evaluation of the Evans blue dye content in retinas of control rats intravitreously injected with PBS or one of the TRPV4 agonists RN1747 (100 µM) and GSK1016790A (100 nM) in the presence and in the absence of vasoinhibins (Vi, 1 µM) for 4 h. Values are mean ± s.e.m. normalized to the control (n = 8–14 per group; *P < 0.05). (**B**) Epifluorescence and confocal images from HMVECs transfected with TRPV4-eGFP, where one can appreciate that TRPV4 is located at the membrane level. Magnification bars, 10 µm. (**C**) Representative measurements of the change in intracellular Ca^2+^ measured by the change in the fluorescence ratio (R340/380) in TRPV4-transfected HMEC with or without 10 µM 4α-PDD and 10 nM vasoinhibins (Vi). (**D**) Corresponding distribution of responding cells (% ± s.e.m.). *P < 0.05. (**E**) MRCEC cells were analyzed for TRPV4 protein. Total β-actin served as loading control. Extracts from three independent MRCEC cell cultures were analyzed (N = 3); MWM, molecular weight markers. (**F**) Time course of TER in MRCEC monolayers cultured in complete medium (control) with or without 10 µM 4α-PDD and 10 nM Vi. n.s., not significant.

**Figure 5:**
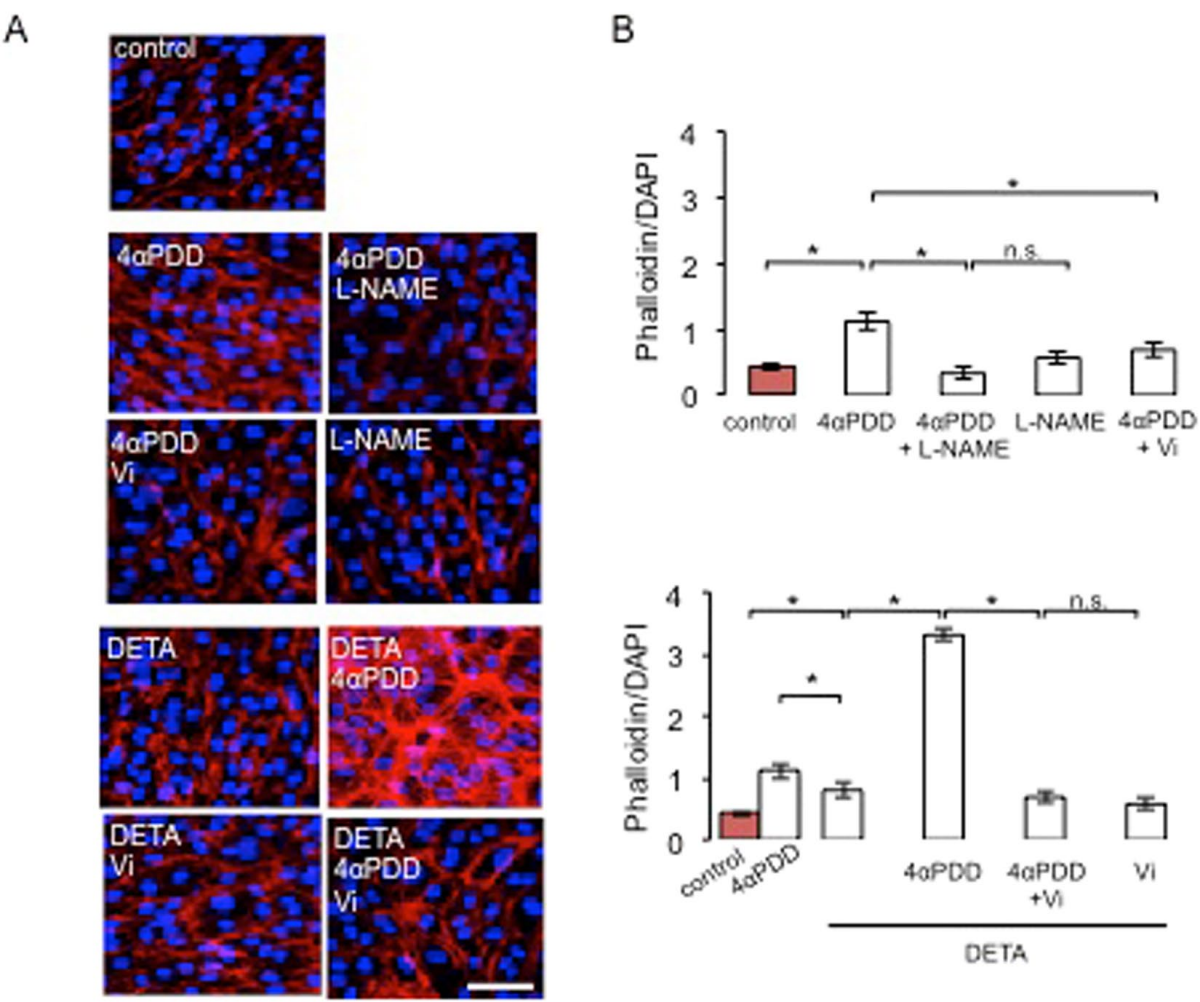
Vasoinhibins block TRPV4-induced actin cytoskeleton redistribution in MRCEC cells in a NO-dependent manner. (**A**) MRCEC were cultured in complete medium (control) with or without 10 µM 4α-PDD and 10 mM L-NAME, and with the NO donor DETANONOate (10 µM) in the presence and in the absence of 4α-PDD and Vi (10 nM) for 5 min (corresponding to the peak TER values measured in panel D in the presence of 4α-PDD), and then actin cytoskeleton (F-actin) distribution was determined using rhodamine-phalloidin. Representative fields are shown. Scale bar, 10 µm. (**B**) Corresponding quantification of phalloidin/DAPI fluorescence. Values are mean ± s.d. (*P < 0.05). n.s., not significant.

Expression levels of endogenous TRPV4 have been shown to be altered in tissues from diabetic rodents, including the retina^41,50,69^. We therefore tested whether vasoinhibins regulate BRB permeability by inhibiting the activation of TRPV4 under non-diabetic conditions. Injection of the TRPV4 agonists RN-1747 and GSK1016790A^39,70^ into adult male Wistar rats caused BRB breakdown, as shown by the retinal accumulation of Evans blue-stained albumin (Fig. 4A). Coinjection of vasoinhibins with either RN-1747 or GSK1016790A reduced the BRB changes induced by both TRPV4 agonists (Fig. 4A). These data show that endogenous TRPV4 channels in rat retina activated with selective TRPV4 agonists, are inhibited by vasoinhibins to maintain BRB permeability. Because most regulatory effects of vasoinhibins and TRPV4 activation on permeability have been defined in microvascular endothelial cells^36,43–46^, and the inner BRB involves the tight junctions between vascular endothelial cells forming retinal capillaries^1,71^, we tested whether vasoinhibins regulate BRB permeability by blocking TRPV4 in microvascular endothelial cells. First, we performed ratiometric Ca^2+^ imaging in TRPV4-transfected human microvascular endothelial cells (HMEC) loaded with Fura-2 AM. TRPV4 expression and distribution at the membrane levels were confirmed^72^ (Fig. 4B, supplemental Fig. 4). Application of the synthetic phorbol ester 4α-phorbol 12-myristate 13-acetate didecanoate (4α-PDD), a TRPV4 agonist that binds directly to the transmembrane region of the protein and selectively activates TRPV4 in non-neuronal cells^23,25,73–78^, induced transient increases in intracellular Ca^2+^ levels in 87 ± 7% of the cells (Fig. 4C). Pretreatment with vasoinhibins inhibited the 4α-PDD-induced Ca^2+^ oscillations (Fig. 4C) and reduced the percentage of HMEC that responded to 4α-PDD to 37 ± 16% (Fig. 4D). Second, in freshly isolated mouse retinal capillary endothelial cells (MRCEC) that were previously shown to present stable resistance over time^13^, Western blot analysis demonstrated expression of TRPV4 (Fig. 4E). In MRCEC, 4α-PDD induced a transient decrease in TER that was maximal at ∼5 min, lasted about 100 sec, and was fully prevented by vasoinhibins (Fig. 4F). Vasoinhibins alone had no effect (Fig. 4F). To complement the TER evaluations, we examined the tight junction-associated actin microfilaments, as they are known to regulate endothelial cell permeability^37,79^. Treatment with 4α-PDD induced stress fiber formation with a uniform polarity (Fig. 5A) that coincided with increased actin fluorescence (Fig. 5B) in MRCEC. These effects were blocked by vasoinhibins (Fig. 5A and B), which were previously shown to have no effect alone^13^. Figure 5B shows quantification of changes in actin fluorescence.

### Vasoinhibins inhibit the TRPV4/Ca^2+^/NO/cytoskeletal reorganization cascade

That TRPV4 C-terminal binding protein microtubule-associated protein 7 has been proposed to link TRPV4 to cytoskeletal microfilaments^80^ suggests that TRPV4 may directly mediate permeability increase. However, it is long known that Ca^2+^ oscillations associate with elevated permeability by targeting many intracellular targets that include intercellular junctions and/or cytoskeletal proteins^37^. Among the major pathways linking cytosolic Ca^2+^ level increases and reorganization of actin cytoskeleton are protein kinase C (PKC), Ca^2+^/calmodulin-dependent protein kinase II (CaMKII), and NADPHoxidase/reactive oxygen species (ROS)^37^. CaMKII stands out because its activation promotes cytoskeletal reorganization through increased nitric oxide (NO) production via endothelial NO synthase (eNOS)-Ser^11,79^ phosphorylation^81^ and CaMKII antagonism prevents TRPV4-mediated effects^82^, including eNOS activation^70^. Vasoinhibins block eNOS activation by abrogating Ca^2+^ transients in endothelial cells^10,14,16^, thereby regulating the BRB^13^. For these reasons, we explored the role of NOS and NO in mediating the inhibition of TRPV4 by vasoinhibins. We observed that coadministration of 4α-PDD and the NOS inhibitor L-NAME^83^ eliminated the 4α-PDD-induced stress fiber formation, but L-NAME alone had no effect (Fig. 5A). Both the NO donor DETANONOate and 4α-PDD increased actin fluorescence (Fig. 5B), but DETANONOate induced the formation of actin stress fibers that show apparently random orientation with respect to each other (Fig. 5A) and 4α-PDD induced a larger increase in actin fluorescence than DETANONOate (Fig. 5B). 4α-PDD and DETANONOate together stimulated actin to levels that overcame their individual effects (Fig. 5B). We then asked whether DETANONOate reverts the action of vasoinhibins in the presence of 4α-PDD. Exogenous NO prevented vasoinhibin-mediated inhibition of 4α-PDD-induced F-actin rearrangement in MRCEC (Fig. 5A). All together, these data suggest that NO partly mediates the 4α-PDD-induced increase in endothelial cell monolayer actin cytoskeleton redistribution, and that vasoinhibins block the NO-dependent component of these actions by acting upstream of NO production, as previously reported^13,14,16^.

## Discussion

Microaneurysms, hemorrhages, and retinal edema in diabetic retinopathy can critically imperil vision, thus greatly contributing to disease-related disability. These retinal changes ultimately reflect BRB breakdown. Our previous work showed that vasoinhibins have significant therapeutic potential for the control of diabetic retinopathy and macular edema since they are natural inhibitors of angiogenesis and BRB breakdown^9–12,49^. Here, we unveil two novel concepts in the pathogenesis of diabetic retinopathy that will further guide us toward rationally-guided new therapies: (i) vasoinhibins can inhibit TRPV4 to maintain BRB and endothelial permeability, (ii) TRPV4 blockage does not seem to contribute to the regulation of BRB and RPE permeability by vasoinhibins under diabetic or hyperglycemic-mimicking conditions; importantly, the TRPV4 antagonist GSK2193874 resolves BRB breakdown associated with diabetes.

### Specificity of TRPV4 detection

TRPV4 is present in mouse retina^18,40^. We detected TRPV4 protein in the inner and outer main components of BRB in adult mice. Our data complemented previous studies^41,42,84^ by detecting TRPV4 protein in ARPE-19 cells and mouse RPE flat mounts, and in primary cultures of mouse retinal microvascular endothelial cells. Because *Trpv4* ^−/−^ retinas lacked immunopositive pattern for TRPV4 when probed with sc-98592 and LS-C94498, both antibodies can be considered specific. The expression pattern revealed by the two antibodies concurred in that cell bodies located in the ganglion cell layer, radial processes of Müller glia, and RPE were immune-positive. However, both antibodies did not recognize a strictly identical TRPV4 pattern: sc-98592 detected immunoreactivity in inner nuclear layer cell bodies that was not present with LS-C94498; sc-98592 immunoreactivity was uniform compared to the LS-C94498 one; and sc-98592 recognized intraretinal capillaries, while LS-C94498 did not. The differential pattern of immunopositive staining for TRPV4 may be due to the recognition of distinct epitopes of the channel protein by the two antibodies. The sc-98592 antibody, also known as H-79, targets an epitope corresponding to amino acids 62–134 mapping near the N-terminus of TRPV4. Immunoblots from several cell types showed a primary band of 132 kDa, and in few cases, a secondary band at ~90 kDa which probably corresponded to N-glycosylated and unglycosylated forms of TRPV4. In our hands, Western blot of ARPE-19 cells and MRCECs showed a unique band at ~100 kDa. In contrast, the anti-TRPV4 LS-C94498 antibody recognized a primary band of 85 kDa and a secondary band at 105 kDa (manufacturer and ref.^40^). This antibody was raised against synthetic peptide from 1^st^ cytoplasmic domain of mouse TRPV4 conjugated to an immunogenic carrier protein. The differences we observed in the expression pattern, in keeping with previous reports, also suggest the existence of TRPV4 isoforms^85–88^.

### TRPV4 as a regulator of BRB?

Although we observed that the TRPV4 antagonists RN1734 and GSK2193874 alone did not alter BRB permeability, intravitreal administration of the TRPV4 agonists RN-1747 and GSK1016790A promoted the accumulation of Evans blue dye in rat retinas, indicating that stimulation of endogenous TRPV4 increases BRB permeability. These data are consistent with the observations that intravenous administration of TRPV4 activators can lead to circulatory collapse, which at least is partly explained by increased capillary permeability and fluid loss in the lungs^46,70,89,90^. Also, 4α-PDD exposure decreases TER in microvascular endothelial cells, including primary culture of retinal capillary endothelial cells, which is in agreement with previous studies^19,34,44,91^. Further analysis should include trpv4^−/−^ mice to define the precise role of endogenous TRPV4 channels in BRB transport.

TRPV4 inhibitors resolve the BRB breakdown associated with streptozotocin-induced diabetes. This effect is new for TRPV4 antagonists in the retina. HC-067047 has been recently shown to reduce brain water content and Evans blue extravasation at 48 h after middle cerebral artery occlusion in mice^92^. In view that *Trpv4* ^−/−^ and wild-type mice treated with a TRPV4 antagonist did not develop insulin resistance under high-fat diet^93^ and that this diet associates with retinal dysfunction^94^, our findings implicate that (i) TRPV4 blockage disables one or more signaling pathways that participate in diabetes-induced alterations of the retinal neurovascular complex^5,66^ and/or that (ii) TRPV4 activity is excessive in the diabetic retina^44^. Observations reporting consistent TRPV4 down-regulation during diabetes challenge this view^41,50,69^. We did not observe any change in TRPV4 protein levels of ARPE-19 cells exposed basolaterally to high glucose. Nevertheless, that high glucose decreased ARPE-19 permeability after 3 days is consistent with the decreased mRNA and protein levels of TRPV4 in retinal bovine capillary endothelial cells (RBCECs) after 3 days of high glucose^41^. Additionally, we found that at day 3, TRPV4 antagonism does not prevent the high glucose-induced increase in ARPE-19 TER, which is consistent with a loss of 4-α PDD-induced Ca^2+^ response in RBCECs under hyperglucemic-mimicking conditions for 3 days^41^. Reduced channel expression is likely to play a role in this effect^41^; however, direct inhibition of TRPV4 channels under high glucose may also contribute to silenced TRPV4 function in ARPE-19 cells.

Reduced TRPV4 channel expression has been suggested as an underlying reason for the dysfunction of vascular function during diabetes, but it may be a compensatory effect in diabetes. TRPV4 antagonism in diabetic rats controled excessive BRB permeability and GSK2193874 protected against the high glucose-induced decrease of ARPE-19 resistance. This further indicates that after 4 weeks of diabetes or high glucose, some TRPV4 is still functional. In agreement with this conclusion, faint yet detectable TRPV4 immunostaining in retinal arterioles and capillaries is seen after 12 weeks of streptozotocin-induced diabetes in rats^41^. We also detected TRPV4 protein in ARPE-19 treated for 28 days with high glucose.

Reduced TRPV4 expression may not always be accompanied by a reduced function because a limited number of functional channels expressed by a cell can completely maintain function. The diabetic milieu modifies many parameters as an early compensatory response, including factors that modulate TRPV4, e.g., generation of arachidonic and epoxyeicosatrienoic acids^95^ (both of them being endogenous metabolites that activate TRPV4), glycosylation levels^96^, and Ca^2+^ homeostasis^97^. Long-term exposure to these mediators associates with diabetic complications that may include the excessive permeability of BRB.

In each tested microvascular beds, the relative sensitivity of TRPV4 to stimulation/inhibition varied; their use therefore only provides qualitative data. The latency (300 sec) and duration (100 sec) of Ca^2+^ response is comparable to the response observed in endothelial cells^89^ and in other cell systems^44,77,98^, and the transient decrease in TER coincides with the rapid (within 5 min) decrease in resistance of mouse mammary gland cell line HC11^44^. In comparing kinetics of 4α-PDD-evoked responses in endothelial cells from similar microvascular beds^24,72,99^ and other cell types^24^, we see that 4α-PDD induced oscillatory [Ca^2+^]_i_ changes and that Ca^2+^ transients preceed and are concomitant to TER decrease, suggesting that both events may be causally linked. This possibility is plausible since several studies established that a rise in cytosolic Ca^2+^ levels is sufficient to activate key signaling pathways that mediate cytoskeletal reorganization (through myosin light chain-dependent contraction) and VE-cadherin disassembly at the adherens junctions, which in turn increase endothelial permeability^100^. Simultaneous recording of [Ca^2**+**^]_i_ and actin dynamics confirmed that TRPV4-induced stress fiber formation takes place during the Ca^2**+**^ elevation^101^. As already mentioned, TRPV4 -mediated Ca^2+^ entry may be sufficient to reorganize actin^80^, but Ca^2+^ is also a potent activator of NOS and we observed that L-NAME blocked the effect of 4α-PDD on actin reorganization. The existence of a TRPV4/Ca^2+^/NO/cytoskeletal reorganization cascade is consistent with studies showing that 4α-PDD increases NO production^36,102–104^ and that responses to TRPV4 depend on NO production^70,105,106^. Furthermore, eNOS has been reported to colocalize with TRPV4^107^, and eNOS expression is up-regulated under mechanic stretch, which associates with TRPV4-gated Ca^2+^ influx^101,108^. Nevertheless, our data showing that both 4α-PDD and DETANONOate increased actin fluorescence have to be taken carefully since DETANONOate appeared to induce the formation of randomly orientated actin stress fibers, while 4α-PDD induced stress fiber formation with a uniform polarity. These differences may be due to pharmacological considerations but they also suggest that 4α-PDD may activate additional pathways than DETANONOate to reorganize actin. The canonical phospholipase A2 and ROS pathways may be involved as previously described^91,101,109^.

Additionally, 4α-PDD induces a transient decrease in TER, as previously reported^44^. Currently, it is unclear as to what ends the TRPV4-mediated regulation of epithelial permeability. The transient decrease in TER may be due to the Ca^2+^-mediated negative feedback inhibition on TRPV4 channels^24,86,110,111^; TRPV4-mediated Ca^2+^ oscillations were previously reported to end rapidly^24^. Also, the surface expression of TRPV4 channels is tightly controlled. TRPV4 proteins may be retrieved from plasma membrane^112^, which would abrogate the signal promoting decrease in TER. It is also important to mention that the large permeability changes observed at days 3 and 28 under high glucose cannot be compared to the acute regulatory effect of TRPV4 stimulation on cell permeability. Usually physiological signals (i.e., bradykinin) transiently regulate barrier permeability. This does not contradict that these same signals induce an exacerbated response under pathological conditions like diabetes, due to alterations in their whole signaling pathway^113,114^ and in the amount and/or nature of TRPV4 endogenous agonist(s), glycosylation levels, and basal levels of Ca^2+^ ^95–97^.

### Molecular crosstalk between TRPV4 and vasoinhibins?

Our data suggest that vasoinhibins under high glucose promote ARPE-19 resistance through a mechanism that is independent from TRPV4 blockage but that under normoglycemic conditions, vasoinhibins can block TRPV4 to maintain BRB and endothelial permeability. So far, vasoinhibins have not been related to any stimuli known to activate TRPV4 (i.e., cell swelling, low pH, mechanical stress, and temperature^115–119^), and TRPV4 has not been related to the few described binding sites for vasoinhibins^120,121^, but vasoinhibins may indirectly regulate TRPV4 by interfering with both receptor-operated Ca^2+^ entry^14,16,17^ and NO production^10,13,14,122^.

On the one hand, vasoinhibins may act upstream of TRPV4 channels. Indeed, vasoinhibins have been demonstrated to prevent activation of phospholipase C by G-protein-coupled receptors, which diminishes intracellular Ca^2+^ release and plasma membrane Ca^2+^ channels activation^16,17^; La^3+^, an inhibitor of receptor-operated Ca^2+^ entry, prevented vasoinhibin effects^16^, and TRPV4 has been demonstrated to participate in the receptor-operated Ca^2+^ entry^25^. On the other hand, vasoinhibins may interfere with TRPV4 activity through Ca^2+^/calmodulin signaling. By abrogating Ca^2+^ transients in endothelial cells, vasoinhibins have been shown to block eNOS activation^14,16^ that mainly depends on Ca^2+^/calmodulin binding^15^. In contrast, Ca^2+^ entry through TRPV4 promotes the formation of Ca^2+^-calmodulin complexes, which binding to TRPV4 enhance channel activity^32,33^.

Vasoinhibins reduce NO production by limiting eNOS activation through Ca^2+^/calmodulin, and also by causing its dephosphorylation by protein phosphatase 2A^10,13,14,122^. NO has been shown to inactivate TRPV4 by two mechanisms: (i) inducing TRPV4 S-nitrosylation on the Cys^8,53^ residue, which reduces the channel sensitivity to 4-αPDD and its interaction with calmodulin^123^, and (ii) increasing cGMP that inhibits TRPV4 by PKG-mediated phosphorylation^124^. In our study, a pretreatment with vasoinhibins would prevent the NO-mediated inhibition of TRPV4. The lack of this negative feedback inhibition would initially increase TRPV4-mediated Ca^2+^ entry and, based on the fact Ca^2+^-mediated negative feedback inhibition on TRPV4 channels has been well documented in vascular endothelial cells^86,110,111^, a similar inhibitory mechanism could occur in our conditions. In this feedback mechanism, Ca^2+^ influx would stimulate a NO-cGMP-PKG and/or NO-S-nitrosylation cascade(s), resulting in inactivation of TRPV4 channels, which would explain the reduced Ca^2+^ transients in response to 4-αPDD in TRPV4-transfected HMECs pretreated with vasoinhibins. We propose that vasoinhibins reduce TRPV4 activity by promoting a microenvironment where the non-capacitative Ca^2+^ entry is depressed and where the Ca^2+^-mediated negative feedback inhibition on TRPV4 channels is enhanced via the abrogation of NO production.

In conclusion, our novel data indicate that TRPV4-signaling can be recruited as a novel target to combat diabetic retinopathy, which is characterized by permeability changes that can be attenuated by endogenous vasoinhibins, which combine synergistically with selective TRPV4 blockers. Interestingly, these two therapeutically-beneficial approaches function via non-overlapping signaling pathways in diabetic conditions. Our findings elevate understanding of the blinding consequences of diabetic retinopathy and pave the way for successfully treating this dreaded and disabling diabetes complication, which is on the rise world-wide.

## Methods

### Reagents

Recombinant human vasoinhibins (corresponding to a 14-kDa fragment of prolactin) used in cell culture experiments were generated by site-directed mutagenesis as previously described^125^. TRPV4 agonists (RN1747, GSK1016790A) and antagonists (RN1734, GSK2193874), mannitol, D-glucose, 4alpha-phorbol-didecanoate (4α-PDD) were purchased from Sigma-Aldrich (St Louis, MO). Rhodamine-phalloidin was purchased from Thermo Fisher Scientific Inc. (Waltham, MA). Anti-TRPV4 (LS-C94498, Lifespan Biosciences, Seattle, WA and sc-98592, Santa Cruz Biotechnology, Dallas, TX) and anti-β-tubulin (ZYMED from Life Technologies; #22833) antibodies were purchased as specified.

### Ethics statement

All experiments were approved by the Bioethics Committee of the Institute of Neurobiology at the National Autonomous University of Mexico (UNAM, protocol #74) and methods were carried out in accordance with the rules and regulations of the Society for Neuroscience: Policies on the Use of Animals and Humans in Neuroscience Research. All efforts were made to minimize the number of animals used and their suffering.

### Animal care and retinal tissue

Male albino rats (Wistar, 250–300 g) and C57BL/6J mice of either sex (4–6 months old) were obtained from commercial suppliers, whereas *Trpv4* ^−/−^ mice were a kind gift from Dr. Wolfgang Liedtke (University). The animals were fed *ad libitum* and reared in normal cyclic light conditions (12 h light: 12 h dark) with an ambient light level of approximately 400 lux. Animals were sacrificed by CO_2_ inhalation and decapitation. Eyes were enucleated and processed by the Evans blue method and immunohistochemistry. Diabetes was induced in Wistar rats with a single intraperitoneal injection of streptozotocin (60 mg/kg)^126^. Animals with glucose levels greater than 250 mg/dl were used 4 weeks after diabetes induction.

### Intravitreal injections

Rats were injected intravitreously as reported^49^. The final injection volume was 5 µl. In one group, one eye was injected with RN1747 (2.4 µg per eye, corresponding to 100 µM as the estimated volume of rat vitreous is 60 µl^127^) or GSK1016790A (1.6 ng, 40 nM) and the contra-lateral eye with RN1747 or GSK1016790A combined with vasoinhibins (1 µg, 1 µM). Controls for PBS and injected protein itself are shown in SI. The control groups consisted of rat eyes injected with vehicle (PBS), and the contralateral eye received RN1747 (100 µM) or GSK1016790A (40 nM). The second group consisted of rat eyes injected with vehicle (PBS), and the contralateral eye received RN1734 (2.1 µg, 100 µM), GSK2193874 (1.7 ng, 40 nM) or vasoinhibins (1 µg, 1 µM).

### Quantification of BRB permeability

The Evans blue dye permeation assay was performed as previously described^55^. It is, however, worth mentioning that in experiments performed in Fig. 4A, Evans blue dye was administered 2 h after the intravitreous injections and left in the circulation for an additional 2 h.

### Histology

See SI.

### Immunohistochemistry

Eyes were fixed in 4% paraformaldehyde. Then, they were washed overnight in 0.1 M PBS, immersed in 15% sucrose at room temperature, oriented and frozen in Tissue-Tek (Sakura Finetek, Torrance, CA), and sectioned (12 µm thick) along the sagittal axis of the eye or dissected for a whole mount of the RPE as described^128^. Retina cryosections were incubated in PBS with 1% SDS for 5 min, rinsed three times with PBS for 5 min, blocked in PBS containing 10% normal goat serum and 0.1% Triton X-100 for 1 h. Double labeling was performed with both anti-TRPV4 and anti-RPE cell specific antibodies [anti-TRPV4 polyclonal sc-98592 antibody, diluted 1:150; anti-TRPV4 LS-C94498 antibody from Lifespan Biosciences, diluted 1:200; anti-RPE65, at 0.08 µg/ml]. After incubation with the primary antibodies, samples were rinsed 3 times in PBS and labeled for 2 h with Alexa-594-conjugated goat anti-rabbit IgG (diluted 1:100), or Alexa-488-goat anti-mouse IgG (diluted 1:500) obtained from Molecular Probes (Eugene, OR). Extensive controls for the anti-TRPV4 LS-C94498 antibody were performed previously^40^, and given that staining with the sc-98592 anti-TRPV4 antibody showed a pattern of TRPV4 immunoreactivity in retina sections similar to that observed with the LS-C94498 antibody (data not shown), we used the sc-98592 antibody. Labeled samples were examined with a laser scanning confocal microscope (Zeiss Axiovert 200 LSM 510 Meta, Carl Zeiss International, Oberkochen, Germany). Images were prepared using the Zeiss LSM Image Examiner.

### ARPE-19 cell cultures

The ARPE-19 human cell line was purchased from ATCC (Number: CRL-2302)^68^ and was grown in Dulbecco’s Modified Eagle’s Medium (DMEM)/nutrient mixture F12 supplemented with 10% fetal bovine serum and 1% penicillin/streptomycin. Cultures were seeded at an initial density of 10^6^ cells and maintained at 37 °C and 5% CO_2_. Cells were used between passages 10–13, reaching approximately 5 weeks in culture.

### Primary cultures of mouse retinal capillary endothelial cells (MRCEC)

We isolated MRCEC from the mouse retina using collagenase digestion and magnetic-activated cell sorting (MACS), ensuring a preparation of >90% purity, as previously described^13^. CD31-positive cells from retinal populations were isolated by MACS as previously described^129^.

### Obtention of TRPV4-transfected human microvascular endothelial cells (HMEC)

HMECs were obtained from derma and were isolated using an anti-CD105 antibody coupled to magnetic beads by magnetic cell sorting using the MACS system (Miltenyi Biotec, Auburn, CA) as previously described^130,131^. HMECs from the derma were immortalized by the infection of primary cultures with a replication-defective adeno-5/SV40 virus as previously described^132,133^. HMECs were grown in complete EndoGRO-MV-VEGF (Millipore) supplemented with 50 μg/mL gentamicin (Cambrex). Cells were used at passages 3 to 15. Periodically, cells were characterized by their morphology and expression of a panel of endothelial antigens such as CD105, CD31, Muc-18 (CD146), CD44, and VEGF receptor 2.

Because HMECs endogenously express low levels of TRPV4^72,134^, they were transfected with the Amaxa Basic Nucleofector Kit for mammalian endothelial cells according to the instructions of the manufacturer using the M-003 program (Lonza) and 2 μg TRPV4-GFP as previously described^72^. Epifluorescence and confocal images from HMVECs transfected with TRPV4-eGFP showed TRPV4 expression (supplemental Fig. 4).

### Immunoblotting

Protein samples from ARPE-19 and MRCEC cultures were resuspended in lysis buffer (0.5% Igepal, 0.1% SDS, 50 mM Tris, 150 mM NaCl, 1 μg/ml aprotinin, and 100 μg/ml PMSF, pH 7.0) and subjected to SDS/PAGE. Total protein (50 μg) was blotted and probed overnight with a 1:500 dilution of the sc-98592 anti-TRPV4 or a 1:1000 dilution of anti-β-actin antibodies. Primary antibodies were detected using an alkaline phosphatase-coupled secondary antibody and a colorimetric detection kit (Bio-Rad).

### Measurement of TER

ARPE-19 cells were seeded on 1.12-cm^2^ Transwell clear polyester membrane inserts (Corning Inc., Corning, NY) with pore sizes of 0.4 µm at an initial density of 150,000 cells per well in DMEM/Nutrient Mixture F-12 Ham medium. After 24 h, TER measured by the EVOM2 Epithelial Voltohmmeter (World Precision Instrument) reached 55 to 75 Ω.cm^2^ and treatments started. They included control (DMEM containing 5.5 mM D-glucose), mannitol (19.5 mM mannitol added in DMEM), and D-glucose (19.5 mM of D-glucose added in DMEM) conditions. Treatments were applied on the lower chamber of membrane inserts, which are in contact with the basolateral side of cultured cells^68^. TER values were expressed as percent of control (complete medium) at time 0.

### Viability assay

The reduction of 3-(4, 5-dimethylthiazolyl-2)-2, 5-diphenyltetrazolium bromide (MTT) was used to assess ARPE-19 viability. Cells were seeded in 96-well collagen-coated flat bottom microculture plates (Corning) at an initial density of 2,500 cells/well and treated for 28 days with the previously described control, mannitol, and D-glucose conditions. Next, cells were incubated with MTT (500 mg/mL, Sigma-Aldrich) at 37 °C for 3 h, and the formazan precipitate was solubilized with 0.4 N HCl containing 10% SDS for 30 min at room temperature and quantified by measuring absorbance at 570 nm.

### Ca^2+^ Measurements using Fura-2 AM

Prior to fluorescence measurements, HMECs were trypsinized and plated onto glass coverslips. The medium was replaced every 48 h. Cells were used 3 days after trypsinization. The culture medium was replaced by HBSS solution containing 142 mM NaCl, 5.6 mM KCl, 1 mM MgCl_2_, 2 mM CaCl_2_, 0.34 mM Na_2_HPO_4_, 0.44 mM KH_2_PO_4_, 10 mmol/L HEPES, and 5.6 mM glucose. The osmolarity and pH of this solution were adjusted to 310 mOsm/L and 7.4, respectively. Dye loading was achieved by transferring the cells into a standard HBSS solution containing 1 μM Fura-2 acetoxymethyl ester (Calbiochem, San Diego, CA) and loaded for 40 min at 37 °C. Subsequently, cells were washed three times with HBSS without dye. Observations were performed at 37 °C on an Eclipse Ti microscope using an S Fluor 20x°—/0.75 NA objective lens (both from Nikon). Images were collected through a Rolera EM-C2 charge-coupled device (CCD) camera (QImaging, Surrey, Canada) controlled with Metafluor software (Molecular Devices, Sunnyvale, CA) and analyzed with Igor Pro software (WaveMetrics Inc., Lake Oswego, OR). >94% of HMECs respond to TRPV4 agonist.

### Filamentous F-Actin staining

TRITC-labeled phalloidin (Molecular Probes) staining was performed and the mean fluorescence intensity ratio (phalloidin/DAPI) was determined as previously described^13^.

### Statistical analysis

All results were replicated in three or more independent experiments. Data are reported as mean ± S.E.M.; all data showed normal distribution or equal variance according to D’Agostino-Pearson omnibus and Levene’s tests, respectively. Differences between two groups were evaluated by a two-tailed Student’s t-test. Comparisons between different groups were determined by ANOVA followed by Bonferroni’s *post-hoc* comparison test (Sigma Stat 7.0, Systat Software Inc., San Jose, CA). Differences in means with *P* < 0.05 were considered statistically significant.

## Electronic supplementary material

Supplementary Information
