# Author Correction: Dual contribution of TRPV4 antagonism in the regulatory effect of vasoinhibins on blood-retinal barrier permeability: diabetic milieu makes a difference

**DOI:** 10.1038/s41598-018-27642-4

**Published:** 2018-06-20

**Authors:** David Arredondo Zamarripa, Ramsés Noguez Imm, Ana María Bautista Cortés, Osvaldo Vázquez Ruíz, Michela Bernardini, Alessandra Fiorio Pla, Dimitra Gkika, Natalia Prevarskaya, Fernando López-Casillas, Wolfgang Liedtke, Carmen Clapp, Stéphanie Thébault

**Affiliations:** 1 Instituto de Neurobiología, Universidad Nacional Autónoma de México (UNAM), Campus UNAM-Juriquilla, 76230 Querétaro, Mexico; 2 Univ. Lille, Inserm U1003 - PHYCEL – Physiologie Cellulaire, F-59000 Lille, France; 3 Department of Life Science and Systems Biology, University of Torino, 10123 Torino, Italy; 4 Instituto de Fisiología Celular, UNAM, C.U., Mexico; 5 Department of Medicine and Neurobiology, and Center for Translational Neuroscience, Duke University Medical Center, Durham, NC 27710 USA

Correction to: *Scientific Reports* 10.1038/s41598-017-13621-8, published online 12 October 2017

This Article contains typographical errors in the Acknowledgements section.

“This study was supported by the UNAM grant IN201814 (ST),”

should read:

“This study was supported by the UNAM grant IN209317 (ST),”

