# Supplementary material for: Dual contribution of TRPV4 antagonism in the regulatory effect of vasoinhibins on blood-retinal barrier permeability: diabetic milieu makes a difference
Source: Sci Rep. 2017 Oct 12;7:13094. doi: 10.1038/s41598-017-13621-8 (PMC5638810; doi:10.1038/s41598-017-13621-8)

Dual contribution of TRPV4 antagonism in the regulatory effect of vasoinhibins on blood-retinal barrier permeability: diabetic milieu makes a difference.

David Arredondo Zamarripa[&], Ramsés Noguez Imm[&], Ana María Bautista Cortés, Osvaldo Vázquez Ruíz, Michela Bernardini, Alessandra Fiorio Pla, Dimitra Gkika, Natalia Prevarskaya, Fernando López-Casillas, Wolfgang Liedtke, Carmen Clapp, and Stéphanie Thebault.

[&] DAZ and RNI contributed equally to this work.

Methods

Histology.

Mouse eyes were fixed by immersion for 48 h at -80°C in 97 % methanol and 3 % acetic acid. After 2 h at room temperature, fixed tissues were rehydrated for 20 min in ethanol:water gradients (from 100 % ethanol to 100 % water; 5 % steps). Eyes were then cryopreserved for 12 h in 10 %, 20 % and 30 % glucose, respectively and embedded in OCT (Tissue-Plus, Fisher Healthcare 4585Ted Pella, Redding, PA). Cryostat was sectioned at 8 μm and mounted on Superfrost slides (Fisher Scientific, Pittsburgh, PA) for hematoxylin and eosin staining, which consisted in tissue immersion for 4 min in hematoxylin Harris, for 1 min in 70 % ethanol and 1 % HCl, for 1 min in 0.5 % ammonia water solution, and for 30 sec in eosin. Each previous step was separated by a washing for 3 min in water. Then, tissue was immersed for 15 sec in 95 % ethanol; for 30 sec, 1 min, and 2 min in 100 % ethanol; and for 2 min in xylene. Samples were mounted using Entellan mounting medium (Merck, Darmstadt, Germany). To quantify the thickness of the retina, images were taken at equivalent retinal eccentricities from the optic nerve head using a microscope with a 20 X objective (Microscope OLYMPUS BX60). Layer thickness was measured in six areas from each retina in three animals per group.

Intravitreal injections.

We analyzed whether injected protein itself can precipitate response in permeability by quantifying Evans blue dye permeation in rats injected with PBS or in which the vitreous was stung, and the contralateral eye received a 5-μl PBS solution containing 1.1 μg or 0.4 ng mannitol, corresponding to the osmolarity controls for the 5-μl PBS solution containing

2.4 μg RN1747 and 1.6 ng GSK1016790A, respectively. The permeability levels were similar in all conditions (supplemental Fig. 2), supporting that PBS is an acceptable control.

Supplemental Figure 1. Representative images of hematoxylin/eosin-stained retinas (*A*) and averaged thickness of each layer of the retina (*B*) of wild-type (trpv4$^{+/+}$) and trpv4$^{-/-}$ mice. Retinal pigment epithelium (RPE), outer segments (OS), outer nuclear layer (ONL), outer limiting membrane (OLM), outer plexiform layer (OPL), inner nuclear layer (INL), inner plexiform layer (IPL), and ganglion cell layer (GCL). White headarrows indicate disturbed OLM in trpv4$^{-/-}$ retinas. Three sections per retina from each of four animals per group were analyzed.

Supplemental Figure 2. Evans blue dye permeation in rats injected with PBS or in which the vitreous was stung, and the contralateral eye received a 5-µl PBS solution containing 1.1 µg or 0.4 ng mannitol, corresponding to the osmolarity controls for the 5-µl PBS solution containing 2.4 µg RN1747 and 1.6 ng GSK1016790A, respectively. Data are expressed in µl plasma x (g retina)$^{-1}$ x h^{-1} ± S.D. n.s., not significant.

Supplemental Figure 3. Separate confocal images of the retina from wild-type and trpv4$^{-/-}$ mice stained with the anti-TRPV4 sc-98592 antibody.

Supplemental Figure 3. Full-size confocal images of transverse sections of the retina from wild-type mice stained for (*A*) RPE-65 and TRPV4 and (*B*) TRPV4 and blood vessel marker, corresponding to panel *B* of Figure 1. Projections in z and merge with DAPI are shown.

Supplemental Figure 4. Epifluorescence images from normal human microvascular ECs (HMVECs) transfected with TRPV4-eGFP to validate TRPV4 expression.

Supplemental Figure 1

A

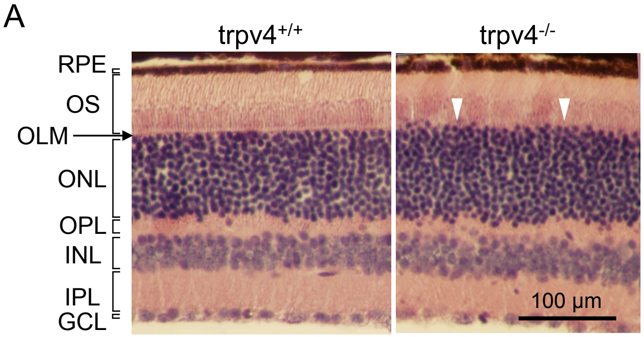

B

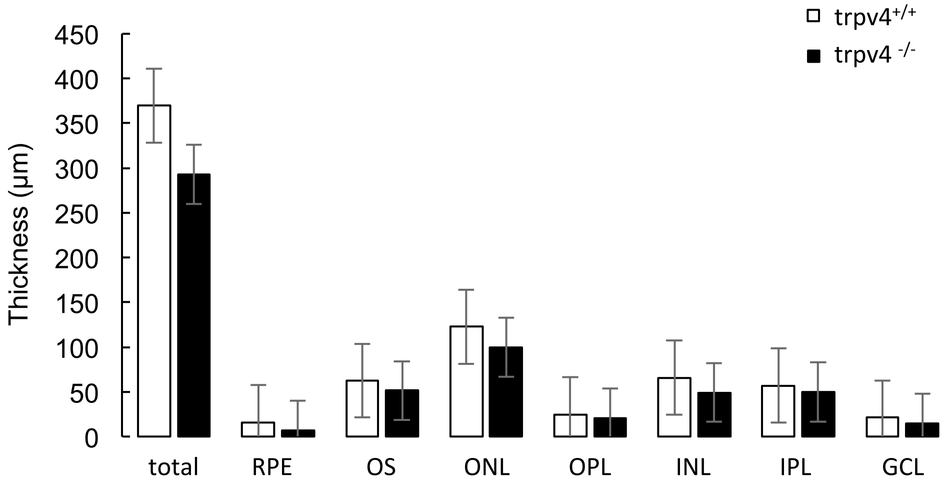

Supplemental Figure 2

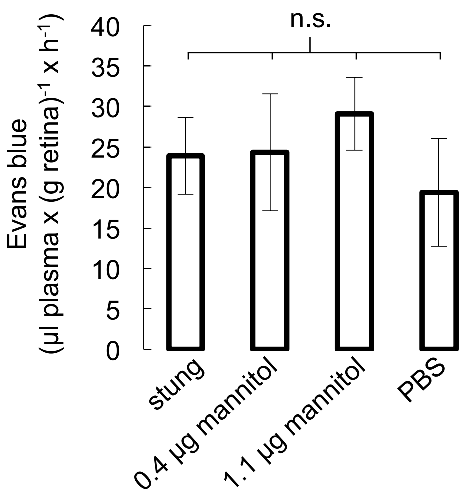

Supplemental Figure 3

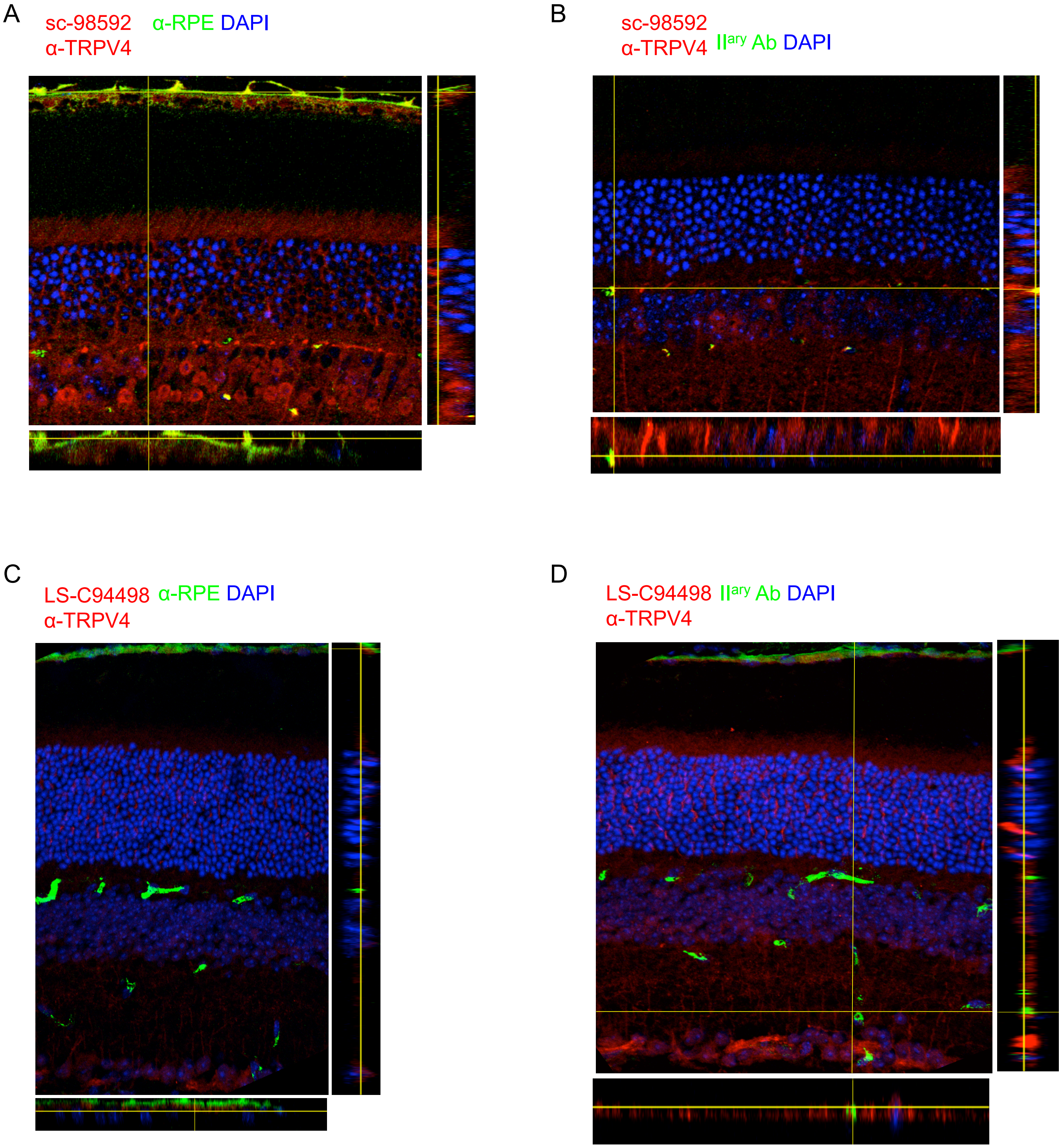

Supplemental Figure 4

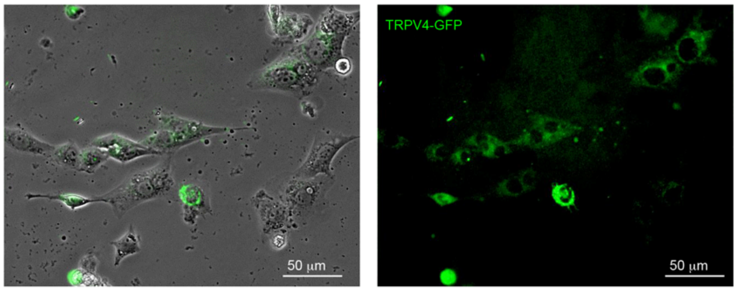

Supplement: Supplementary file 1 — Supplementary Information [file 41598_2017_13621_MOESM1_ESM.pdf]
